# An Ethnographic Study Concerning the Implementation of Education on Ageing for Older Adults with Mild Intellectual Disability: The Perspective of the Educators

**DOI:** 10.3390/ijerph21070953

**Published:** 2024-07-21

**Authors:** Marianne Holmgren, Gerd Ahlström

**Affiliations:** Department of Health Sciences, Applied Gerontology, Faculty of Medicine, Lund University, P.O. Box 117, SE-221 00 Lund, Sweden; marianne.holmgren@med.lu.se

**Keywords:** ageing, education, ethnographic study, health promotion, healthy ageing, learning disability, implementation, mild intellectual disability, pedagogic implementation

## Abstract

Despite the fact that longevity in people with intellectual disability has increased at least as much as in the general population, there is a dearth of interventions related to ageing for these older people. Therefore, this study investigated educators’ implementation strategies in a new tailor-designed educational intervention with the goal of supporting the process of ageing for people with mild intellectual disability. An ethnographic research design was employed, including participant observations, field notes, and 15 ad hoc interviews with educators, spread over two years in four towns. The strategies used for facilitating learning about ageing were expressed in the two themes *promoting social togetherness through everyone’s participation* and *learning together and from each other through recognition and consolidation*. These strategies were applied to create a learning environment characterised by a good atmosphere and respectful interaction. Learning together involved consolidation through repetition, group discussions, the use of visual learning materials, and study visits. This new educational intervention about ageing is promising, but less resource-intensive interventions should also be developed and preferably integrated into the disability service. Before concluding whether this education supports the ageing process, it needs to be evaluated from the perspective of people with intellectual disability.

## 1. Introduction

The success of a new intervention in achieving its goal requires knowledge of the specific context and in-depth knowledge of the current target group of the intervention on the part of the programme designer. If this is the case, tailor-made designs, strategies, and content can be developed, as is frequently recommended in the literature [1,2]. At this stage, user involvement is recommended as a key component for tailoring interventions and improving adaptation to contextual needs and preferences [3,4,5]. Another key component in the success or failure of an intervention is the implementers, who need to be able to carry the specific context and local needs. Implementers need to find the aims, strategies, and materials of the intervention acceptable, useful, and supportive for their practice, otherwise their influence on the intervention may contribute to its failure [6,7]. This study of educators as implementers is the first part of an evaluation of a newly designed educational intervention on ageing for people with intellectual disability.

Previous studies that have investigated the ageing experience of people with intellectual disability have highlighted the need for support in the ageing process, including social networks, accommodations, and activities [8,9,10,11]. Ageing is a natural process with inevitable challenges and life transitions that go beyond healthcare needs, requiring support strategies to cope with these challenges [12]. Supporting ageing involves providing opportunities and an enabling environment to facilitate the maintenance of wellbeing, independence, self-determination, and active engagement in life [12,13]. However, there is a dearth of ageing interventions for older people with intellectual disability in the literature [14], despite longevity in individuals with intellectual disability having increased at least as much as in the general population [15,16,17]. In recent decades, a large body of epidemiological research has been published, showing that people with intellectual disability over the age of 50 years have poorer mental and physical health than age-matched people without such disability [18,19,20,21,22,23]. This points to the urgency of focusing on ageing interventions for older people with intellectual disability [24,25] in order to avoid unnecessary worries and anxiety [9,10,26].

Intellectual disability is clinically recognised during early infancy and childhood, and it is diagnosed before the age of 22 years [27,28]. Diagnoses are divided into mild, moderate, severe, or profound. Such intellectual disability is characterised by significant limitations in both intellectual functioning (learning, problem solving, and judgement) and adaptive behaviour in activities in daily life, such as communication skills and social participation. This study focuses on education for people with mild intellectual disability, which means that they have an intelligence quotient (IQ) of less than 70 but have more abilities than people with moderate, severe, or profound intellectual disability [29,30].

Studies have found that most people with intellectual disability are able to incorporate important health knowledge [14,31], such as the meaning of the ageing process, but may require adapted pedagogical strategies. Santos et al. [14] presented a healthy ageing perspective in their systematic review; they recommended that healthy ageing interventions should take into account the unique preferences of people with intellectual disability [14] in order to develop support according to their needs during the transition to old age [32]. Without support, this transition can negatively affect their wellbeing and quality of life due to cognitive limitations in understanding abstract phenomena [27,33]. The authors’ review was based on 23 articles describing a variety of aspects of healthy ageing: ten were about physical activity and health nutrition, six were about health education and health screening, three were about social inclusion and community participation, and four were about multiple components. The six interventions concerning health education and health screening addressed physical activity and fitness, nutrition and diet, health screening knowledge, and behaviour change techniques [14,34]. The review confirmed that interventions for older people with intellectual disability are scarce, sporadic, and do not focus on ageing. Thus, there is a need for further development of ageing interventions for people with intellectual disability.

Against this background, an intervention named “Good Life in Old Age” was developed and implemented in an attempt to reduce the knowledge gap in the literature on education that supports the process of ageing among people with mild intellectual disability. The intervention was developed from group sessions and individual interviews with older people with intellectual disability in order to ascertain their preferences about ageing (user involvement), as well as from a research study involving interviews with 26 people with mild intellectual disability [9]. This resulted in the implementation of a long-term educational intervention for people in this group. The education consisted of adapted pedagogic strategies for intellectual disability to make the meaning of ageing more concrete and understandable through study visits in the society, using visual materials such as pictures and videos as well as storytelling.

According to Shek and Ma [6], it is important to examine the implementers’ view of an intervention as part of an evaluation because they have first-hand experience of the process of implementing the intervention. Their views may be more accurate in some respects than those of the intervention participants in terms of their professionalism and knowledge. They can also provide a transparent and accurate picture of the implementation quality and provide insights into the context in which the programme operates. When an evaluation is based on different data sources, collecting the views of the programme implementers can increase the credibility of the evaluation according to the principle of triangulation [6,7]. To better understand the findings of future evaluative study with people with intellectual disability after the educational intervention, we therefore investigated the educators’ perspective of implementing the intervention “Good Life in Old Age”. Thus, the aim of this study was to investigate the educators’ implementation strategies in the new educational interventions with the goal of supporting the process of ageing for people with mild intellectual disability.

## 2. Materials and Methods

This study employed an ethnographic research design with participant observations, interviews, and field notes. The ethnographic method was used to attain a deeper understanding of the strategies applied by educators (in the role of course leaders) concerned with educating people with mild intellectual disability on the subject of ageing. Ethnography combines the insider’s perspective (emic) and the researcher’s perspective (etic) to achieve a theoretical description of the social world and draw a more abstract conclusion about it. The three datasets (participant observations, interviews, and field notes) were integrated into a comprehensive description of the educators’ facilitating behaviour, influenced by the cultures or subcultures in which they live [35,36].

### 2.1. Educational Intervention

#### 2.1.1. The Healthy Ageing Intervention “Good Life in Old Age”

The goal of the intervention was, firstly, that the persons with mild intellectual disability received support for their own ageing through knowledge-based education and, secondly, to help them become senior experts in healthy ageing issues, ready to share their knowledge about such issues with politicians, civil servants, and journalists. The dissemination of knowledge can play an important role in improving social services and healthcare for people with intellectual disability. The overall vision behind these goals was that good ageing for the people themselves, as well as the opportunity to have an influence in society, is in agreement with the WHO definition of healthy ageing: “the process of developing and maintaining the functional ability that enables wellbeing in older age” [12,37]. Cosco et al. [38,39,40] analysed the many definitions of healthy ageing, such as successful and active ageing. The intervention “Good Life in Old Age” equates healthy ageing with active ageing.

This healthy ageing intervention was carried out in four towns spread out over Sweden, initiated by an experienced former administrator for ageing issues at the National Association for People with Intellectual Disability. Responsible for its management were this experienced leader together with a researcher and teacher in special education. Initially, the content of the intervention was developed in collaboration with persons with intellectual disability from the local associations of the National Association for People with Intellectual Disability. This process of user involvement consisted of group sessions and individual interviews, where participants shared their thoughts about matters of importance as older people. This contributed to a two-year-long education that was tailored to the needs of older people with intellectual disability, taking into account their unique preferences. The educational content was compiled into comprehensive study guides (30–40 pages for each of the four semesters, written in Swedish), which were used by the course leaders during the course.

The overall content of the course included ageing, accommodation, activity, and participation. In addition to teaching, the programme included several study visits, visits by experts, group discussions, and continuous follow-up of the previous sessions; it started in autumn 2021, with course sessions held once a week in four towns, lasting for two years (see the schedule in Table 1). Each session was 3 h long, with a break in the middle.

The course leaders followed the study guide, implementing course schedules of 10–15 sessions per term. In addition, the project management team visited each course 2–3 times per term in each town to monitor and support the implementation of the healthy ageing educational intervention. The two authors evaluated the intervention and were not engaged in the implementation of the education.

#### 2.1.2. Course Members of the Educational Intervention

People with mild intellectual disability were chosen because this target group was expected to benefit most from this educational intervention. The plan was to have 10 course members in four towns (totalling *n* = 40); however, recruitment took time, and it was difficult to find persons who fulfilled the inclusion criteria of being ≥50 years old with mild intellectual disability and who were willing to participate in the weekly course over the two years. During the recruitment phase, the course leaders, or care staff, or a coordinator for the Adult Educational Association contacted persons who fulfilled the inclusion criteria. The persons who initiated contact were well known by the eligible people from previous teaching settings, or from support and service to those people in daily living. Twenty-six people started the course, and information on these participants was provided in a recent paper [9]. Six members left the course, one died, and the others chose to prioritise other activities. Four members joined after the course had started, having been recommended by course members who already attended the education sessions.

### 2.2. The Ethnographic Study

#### 2.2.1. Sampling of the Study Participants

This study recruited persons suitable for serving as course leaders in the four selected towns across Sweden. Eligible course leaders needed to have experience working with people with intellectual disability and be recognised for their competence by the local branches of the National Association for People with Intellectual Disability. Two prospective course leaders were contacted by these organisations in each of the four towns. These eight persons, all women, agreed to participate; they were aged 45–71 years (mean age: 63.5 years). The range of experience of working with people with intellectual disability was 6–50 years (mean: 23.0 years), and the experience as a course leader varied from 0 to 46 years (mean: 15.5 years). In each town, at least one course leader had prior experience as a course leader. Three of the eight course leaders had experience leading a course on ageing for people in the general population.

Three course leaders ended their assignments before the end of the two-year course. Two resigned after one and three terms, respectively, for personal reasons, and one resigned after two terms because of retirement. One of the three was replaced by another course leader with similar experience, while the other two were not replaced.

#### 2.2.2. Data Collection

Data collection was carried out one day during each of the four terms in each town by one of the two authors. The authors used a manual for participant observation in order to understand what happened in the sessions, what sort of atmosphere there was, and what changes occured over time (Table 2). There was 45 h of participant observations during the two-year intervention, spread over 15 days.

The first author attended four sessions in one town and three sessions in another town. The second author participated in four sessions in each of the other two towns. Following each observation, an ad hoc interview was conducted with the course leaders to determine their perceptions of applied strategies that day and any observed changes in the group dynamics. These interviews were digitally recorded and transcribed verbatim. Field notes were also written after each observation.

#### 2.2.3. Data Analysis

A qualitative inductive thematic analysis was applied [41,42] by both authors. The authors started by listening independently to the digitally recorded interviews in order to obtain a naive understanding of the meaning and nuances in the course leaders’ experiences. Memos were written to capture meaningful segments of the text. The transcribed field notes from the observations and the ad hoc interviews were integrated into a single text and read as a whole to obtain a deeper understanding of the text. Through a combination of listening, reading field notes, interviews, and memos, in conjunction with discussions between the authors, preliminary categories indicative of core content emerged. Subsequently, the text was coded under these preliminary categories using NVivo software (version 10) [43] by the first author. The coded text was interpreted more deeply by each author reading it back and forth. The preliminary categories and their connected text were discussed by the two authors at several meetings, and sub-themes and themes were developed and refined.

## 3. Results

Overall, the data from participant observation, field notes, and interviews together showed that the course leaders perceived the aim of the intervention to be relevant and important for them to implement in the education. Perceptions of the study guides varied between course leaders; most found them to be clear and very useful. These course leaders followed the study guides closely and found them to be very supportive and invaluable to their practice. In these cases, they worked according to the suggested plan of each session (Table 1), with materials, study visits, learning tools such as film and video, and questions for group discussions. The study guides saved these course leaders a lot of time in preparation before each course session. Other course leaders saw the content of the study guides more as recommendations to be followed on a voluntary basis. The number of study visits varied between the groups, and this meant that their own local familiarity and network in the local community gave them the opportunity to arrange relevant study visits for learning about ageing for the course members. The supportive role of the project management for the course leaders was perceived as a resource and security, while it was unclear what support they could expect. The course leaders’ strategies to implement the study guides for teaching about ageing are presented in terms of two main themes: promoting social togetherness through everyone’s participation and learning together and from each other through recognition and consolidation, with their corresponding sub-themes (see Table 3). Promoting social togetherness was characterised by the great respectfulness shown by the course leaders, fostering positive group dynamics. This created a good atmosphere, and there was good interaction among the members of the group, leading to enhanced mutual understanding and respect. Socialisation was strengthened through coffee breaks, as was clearly observed by the researchers and highlighted by the course leaders in the interviews. Learning together shaped the learning environment, where both course leaders and course members learned together and from each other. Course leaders were responsive to the needs of the course members on the particular day, whereby the latter became more receptive to the course content, which facilitated learning. The best way to consolidate the course members’ knowledge was by repetition.

### 3.1. Promoting Social Togetherness through Everyone’s Participation

#### 3.1.1. Respectful Meetings in Close-Knit Groups

The course leaders promoted interaction among the course members by leading conversations with respect. Every effort was made to ensure that all course members actively participated in the sessions and were stimulated to participate in the decision making and show respect for one another’s individual autonomy. The familiarity among most course members prior to the sessions was advantageous. Some initial turbulence occurred in the groups during the first term, such as when someone dropped out and a new course member arrived. From the second term onwards, a sense of fellowship and respectful interaction was established, creating a pleasant atmosphere where everyone had the opportunity to talk and members respected one another’s space.

The interaction in each group evolved and deepened throughout the course, which led to enhanced understanding, greater mutual respect, and greater social togetherness. The growing bonds among course members fostered an increasingly strong sense of security, resulting in harmonious group dynamics described as “cosy and nice” by the course leaders. This harmony, respect, and sense of security encouraged course members to open up and share their feelings.

The strong cohesion and security within the groups allowed new course members to integrate into the group as the intervention progressed. When a new course member was to be recruited, the course leaders discussed it with the group, who usually said that they would accept the new course member if they already knew the person. It is probably because of this that the group dynamics did not change, despite the inclusion of new course members.

*Two people have dropped out and one person joined the group right at the end of last term, but the group have coped with the changes very well indeed. It’s a stable group and they’ve got enormous trust in one another. And I don’t think anyone lets anything that’s said go further, because we’ve been clear about that: what we say stays within these four walls. I really do feel there’s a give and take in this group, based on trust. There’s cohesion. I think some of them were acquainted with each other before, including the person who joined the group at the end of last term. I’ve in fact realised that the only one that didn’t know her was me. Otherwise, it can be difficult to bring in a new person. But the fact that the others knew her meant that she very quickly blended in. The group cohesion being so good, she was soon at home*.

(Interview with course leader)

#### 3.1.2. Reinforcing Social Coherence

A valuable part of the course was the coffee break, with fruit or biscuits, which was unexpected by the course members but much appreciated. It was clear both to the course leaders and to the researchers that this break played an important role with regard to socialising; it was a welcome break and gave the course members the opportunity to talk about whatever they wanted to. The social togetherness of the intervention was very important. The course leaders were constantly concerned with the course members’ wellbeing and warmly shared in their joyfulness.

*…and then it was time for coffee, and one of the course members had baked scones and a banana cake. She did the serving too—spread the table, poured out the coffee and served the scones and cake. They talked to one another a lot in the coffee break, and there was a very pleasant and happy atmosphere*.

(Observation note)

### 3.2. Learning Together and from Each Other through Recognition and Consolidation

#### 3.2.1. Responsiveness Is the Path to Knowledge

The sessions were characterised by great responsiveness from the course leaders. The course leaders saw themselves as open and responsive to what was important to the course members. They began the sessions by listening to the course members and capturing what was important to them on that particular day, which was often about inequalities in disability services and everyday life. They believed that it was important to give the course members time and space to talk about what was on their minds at that moment, albeit constantly keeping in mind the objective of the intervention. From what the course members said at the beginning of the sessions, the course leaders were able to steer the discussion towards the content of the course on ageing; they believed that learning would be facilitated if the course members were allowed to begin by talking about what was most important in their minds at that moment. The course leaders spoke of the joy that they experienced in following each person’s growth as a group member, as well as the whole group’s growing collaboration.

Most of the course leaders appreciated and prepared the course sessions based on the solid, detailed, user-friendly study guides. However, sometimes, adjustments had to be made to accommodate the varying needs of the course members. In such cases, the course leaders found great benefits in applying the knowledge and experience that they had gained from the planning and delivery of previous courses, as well as from their previous work with people with intellectual disability. This meant that they were able to use the study guide to their advantage on different occasions.

*It’s a question of getting a good discussion going. I often use conversation cards—a card’s drawn and I start a sentence and they go on from there. But if they’ve got their own idea about what the subject for discussion should be, I’m glad to go along with it. It means, of course, that we’re not always in line with what the study guide says—but sooner or later we get on to the subject of ageing. You asked whether this business of ageing is their main focus. It hardly ever is*.

(Interview with course leader)

Another example of the course leaders’ responsiveness was seen in their adaptation to conversations about grief and death. Such conversations arose both spontaneously and planned, which meant that the leaders’ preparation for the conversations about grief and death varied. Situations that arose regarding the death of a member or a relative led to conversations about grief and death, which required flexibility on the part of the leaders. The conversations about death and grief were thus something that they could not postpone. It was emotionally challenging for the leaders to talk about grief and death when they themselves were weighed down by grief, while it was part of both the members’ and their own processing. Spontaneous conversations about grief and death also arose when the leaders understood that there was a need and then gave the members time and space. Such conversations could be about the members’ long lives with bullying or dead parents. Even the planned sessions addressing the topics of grief and death were a challenge for the course leaders, as they were concerned about evoking strong emotions in the course members. While they felt that they had to be prepared to deal with this topic, they were unsure of their own ability to do so; therefore, they enlisted people who were more at home with such discussions, such as deacons and funeral home workers.

*…then I also want to bring in a priest who really has the focus to talk about life and death and how to plan. What happens before a funeral, what happens after. But I don’t have everyone with me, I have a woman who is so incredibly afraid of death… And that’s exactly why I think this is so necessary that we bring in a priest who talks. Try as number one to calm her down, but also that everyone can ventilate, because there were several here who said “Yes, but it sounds great, we would like that”*.

(Interview with one course leader)

#### 3.2.2. The Right to Be Heard and to Have an Influence

The course leaders showed genuine respect for the course members, letting them be involved in the formation of the course and encouraging them to speak up. By encouraging and strengthening the members in terms of their right to age like everyone else, they provided them with better conditions to achieve healthy ageing. The course leaders knew that this was a challenge because this group is not included at all in society. Involving them in meaningful activities and satisfactory accommodation may be one part of healthy ageing, according to the course leaders. The course members’ unique experiences from living a long life with an intellectual disability were shared with the course leaders. Thus, the course leaders and members all learned together.

*… the main thing is that we both have a genuine interest in hearing what they have to say. It’s not a question of us teaching them but of all of us learning together…. Yes, you’ve got to adjust your way of explaining things and build on what they already know about ageing and add a few facts… and have respect for the person you’re working with…. Then I suppose it must be an advantage that we know our course members, some of them pretty well*.

(Interview with two course leaders)

The course leaders sought to make the course members aware that they could have an influence; they did so by heeding the course members’ wishes. If, for instance, the course members expressed a desire to visit a certain place or meet a certain person, the course leaders tried to comply with this as far as possible.

*Yes, and then X said right away, “We must go there on a study visit!” Then we’ve got to see if we can squeeze it in somewhere, this term or next term or the last one…. They’ve asked about it several times. “We want to go there!” To meet some clergyman or welfare worker or… They’re ever so interested, they really are. They’re full of expectancy*.

(Interview with two course leaders)

One aspect of ensuring the right to be heard and have an influence was to allow all course members time to talk without interruption. In the beginning, a cuddly toy was used; to be allowed to talk, a member needed to receive it from the person who last talked. However, after a term, there was discussion as to whether this was an effective method, and it was gradually replaced by the traditional method of raising hands. Most of the time, the latter method worked well. Even though the course members showed respect for one another, they sometimes had difficulty waiting for their turn to talk. This resulted in increased chatting. The course leaders handled these situations by gently guiding the course members back to the taking of turns.

#### 3.2.3. Facilitated Responsibility for Knowledge Acquisition

As part of the course, the course leaders supported the course members in devising questions to present to authorities, associations, and accommodation staff; they made sure that the course members felt safe asking their questions and that they could use familiar language. The individual course members “owned” their questions, and it was important that the original questioner was the one who asked them during visits. When some questions were not suitable for the current visit, they were saved in a question bank to be used at a later time.

*The way we work in this course is that we keep to the same structure of questions all the time so that the course members will feel more secure in this respect. Their questions get more power, they feel that this is my question, I own it. And sometimes perhaps there’s a question that’s not suitable for this particular visit, so it’s saved for later. Some of the questions that come up are more political or are to be directed to a particular official. Then I put them in a little kitty, as I call it. So we’re always collecting our own material*.

(Interview with course leader)

The course leaders said that repetition was the key to consolidating the course members’ knowledge, along with visualisation and giving them plenty of time to express their thoughts and opinions, and to discuss among themselves what they had experienced, such as issues that had been raised during the study visits. The study visits were important, especially when it came to accommodation. Through meeting people in new environments during the course, especially when visiting different types of accommodation, the course leaders perceived that the course members became more receptive to new knowledge. When it came to politicians and civil servants, the course leaders thought that it would work equally well if the person came to the location where the course session took place.

*It’s good that we plan the questions together. Previously, we only had handwritten questions for the next study visit. Now, we’ve adopted the idea of showing their questions on a big screen, it makes things a little slower and they have time to take in the questions one by one. Yes, I really think that’s better. They’re more active, go through it a second time. Yes, it’s a kind of repetition. We all together write down the questions, the answers and their reflections… So we take it step by step, so that it doesn’t go too fast. This gives them the chance to think back, think about where they’ve been and what they’ve experienced*.

(Interview with two course leaders)

## 4. Discussion

### 4.1. Results Discussion

The main findings mostly expressed a successful implementation of the tools for learning ageing from the study guides. The course leaders, as implementers, had long experience in communicating with and understanding the needs of people with mild intellectual disability. This may explain why they found the aims to be relevant and the learning tools of the tailored educational intervention to be supportive both for themselves and for the future improvement of older people with such disability. The course leaders’ implemented strategies were *promoting social togetherness through everyone’s participation* and *learning together and from each other through recognition and consolidation*. These implementation strategies illustrate that this ageing intervention can be placed in the categories of social inclusion and community participation, in accordance with Santos et al.’s systematic review of healthy ageing [14]. The importance of promoting social togetherness as a way of preventing social isolation has been explained in previous studies. The inadequacy of social networks and lower levels of participation in society characterise the life situations of people with intellectual disability [44,45,46]. In addition, social isolation and loneliness are described as risk factors for poor mental and physical health in the general population [47].

In the present study, the course leaders promoted social togetherness by facilitating the interaction between the course members, which they did by leading the conversation respectfully; they ensured that all of the course members could be active, show respect for one another’s individual autonomy, and be safe. A positive atmosphere has been described as a facilitating strategy to ensure the progress of the learning process [48]. Similar endeavours have been found in previous studies. Meys et al. [49] conducted semi-structured interviews with adults with intellectual disability/autism spectrum disorder, professionals, and network members; they found that getting to know the person with a disability well, working with an empowering attitude, and being a safety net were important factors for strengthening social relationships [49], which was consistent with the attitudes of the course leaders in our study. Moreover, the course leaders’ strengthening of social togetherness and safe learning in the present study is mirrored in a study on leaders by Wilson et al. [50]. Persons with intellectual disability were invited to form a group focused on social togetherness, where the leader’s assignment was to integrate and lead the participants in shared activities. The group was based on people having shared interests and needing an expanded social network; they wished to partake in social togetherness but often lacked the skills to do so because of their intellectual disability. Ten participants were individually interviewed about their experiences of participating in this group. The results also showed that with appropriate and targeted support adults with intellectual disability experience richer and healthier lives. The leaders made it easier for the members of the group to take part in activities together, thereby contributing to the socialisation of persons with intellectual disability. The group grew and, after five years, it became part of an existing recreational service.

Furthermore, with their competence from earlier work of supporting persons with intellectual disability, the course leaders in the present study used strategies such as responsiveness and respect to achieve social togetherness. This is in line with the study by Bigby and Wiesel [51], who emphasised that the leader’s competence is crucial in social meetings for people with intellectual disability; they highlighted both involvement and a common purpose for participation as important when it comes to achieving social togetherness. This common purpose, in the case of our study, was education that supports the process of ageing. The attitude of respectfulness and establishment of security by the leaders described in our study emphasises the importance of leadership support for course leaders in promoting social togetherness for people with intellectual disability; in our study, this support was provided by the project management board. This dimension should therefore neither be forgotten nor underestimated when planning an ageing intervention for individuals with intellectual disability.

The strategy learning together and from each other through recognition and consolidation in the present study meant that the course leaders needed to give the course members time and space to talk about their daily issues and topics of interest to them, which often involved inequalities. This strategy was necessary for the course leaders to capture the course members’ attention in preparation for steering the conversation towards ageing. Inequities are sustained throughout life and are seen to increase with age [12]. Disability conditions from an early age lead to inequality in health throughout life. To support healthy ageing, attention must be directed to those who are most disadvantaged, thereby reducing inequities [12]. The support that the leaders in the present study provided through shared learning about ageing with people with intellectual disability can enable equitable and healthy ageing. This result is consistent with the findings of the WHO [12], who highlighted that supporting continued personal development—mentally, physically, socially, and emotionally—allows older people to take control of their lives, make active choices, and continue to learn, grow, and make decisions that are best for them. Older people’s learning provides them with the skills to take care of their health, and they experience heightened self-confidence and self-actualisation. These skills keep older people more involved in community activities [12]. Allowing the members in this intervention to share their unique experiences from living a long life with intellectual disability resulted in common learning about the ageing process, which can be expected to facilitate the progression of the healthy ageing intervention. In addition, to strengthen the implementation of healthy ageing, education is required for professionals and the general public [52]. Education can empower people and change the ways in which they think about ageing. Above all, the development of an age-friendly environment that is accessible, equitable, inclusive, safe, secure, and supportive is needed.

In a previous study, learning together was implemented in 16 courses, each consisting of ten weekly sessions with people with intellectual disability and conducted by eight different course leaders [53]. Despite the differences in time period and the number of courses, there were several similarities to our study. The course leaders perceived that learning together offered good opportunities to increase health literacy through group activities adapted to individual characteristics. Learning by doing, in conjunction with study visits, was seen as being especially appropriate for persons with intellectual disability, since this form of learning is visual and concrete. As in the present intervention, repetition was highlighted as valuable; it provided the participants with a feeling of trust and security [53]. There is a need to understand the culture of learning; we need to understand the relationship between how people learn and the contexts or environments in which they learn [54].

In this study, learning together about healthy ageing also meant sharing experiences about grief and death. The leaders felt that they had to be prepared to deal with these existential topics, which they usually do not work with. Two interview studies found that staff working in disability services perceived it as extremely difficult to encounter people with intellectual disability coping with grief and death [55,56], since they lacked the necessary knowledge and training. They used strategies to avoid open communication about death, such as using euphemisms, in order to protect both themselves and those with intellectual disability [56]. Furthermore, in a study carried out by Fernández-Ávalos et al. [57], persons with mild and moderate intellectual disability having to cope with grief and death were allocated to an intervention group or a control group; the 20 individuals who were part of the intervention group were interviewed after four sessions of an educational intervention performed by a psychologist, dealing with strategies for coping with feelings of loss and grief. Although four sessions was probably not enough, a reduction in intense grief was seen [57]. Because of the group members’ difficulties in understanding abstract phenomena (such as the meanings of ageing, grief, and death) due to their disability [27,33], the leaders’ strategies must take into account these prerequisites for learning. Given the increasing number of ageing people with intellectual disability, grief and death need to be given more attention in future healthy ageing interventions.

The number of people with intellectual disability in each town’s group did not reach the planned ten, but the course leaders were satisfied with fewer course members because it made it easier to achieve a sense of group community and for everyone to have a say. As replacements, the course members suggested other people they knew with intellectual disability, which helped to maintain the positive group dynamics. As the course continued, the group became more and more close-knit, and when a new member was recruited, it was important for the course leader to ensure that this person blended in. Bringing together ageing people with intellectual disability in a learning situation requires sensitivity and reflection.

The results of the implementation indicate that the project management needs to be more responsive if a course leader does not use the comprehensive study guides for each semester as intended, in order to avoid unequal educational content between the groups, and this can be ensured by more frequent follow-ups by the project management. Another conclusion from the implementation is that the education is too resource-intensive and is delivered over too long a period of time, as evidenced by the changes in course leaders and course members, as well as by the difficulties in recruiting people with mild intellectual disability to the intervention, despite this group being the largest compared to moderate and severe intellectual disability [58]. One suggestion is to integrate the intervention into the regular disability service, where two experienced staff members are assigned as course leaders to use the study guides developed for “Good Life in Old Age”. Preferably, the intervention would cover more than one disability service in order to save resources. One of the two course leaders should have competence and experience in elderly care [24].

### 4.2. Methodological Considerations

Some methodological considerations need to be highlighted in this research. A weakness is that the educational intervention (and, therefore, the results of this study) cannot be transferred to people with moderate or profound intellectual disability without suitable adaptation. In addition, the local branches of the National Association for People with Intellectual Disability hand-picked the course leaders, which may have affected the credibility of the results. The sense of being chosen may explain why they had a positive attitude towards the intervention and were very pleased to be appointed as course leaders. Another possible reason for this positive attitude is that they knew how great the resources put into this intervention were and greatly appreciated that these resources were directed towards such a vulnerable group. A further factor is that the project leader was well known to some of them, though this may have been negative at the same time, in the sense of hindering course leaders from raising criticism in the interviews.

The results of this ethnographic study contribute knowledge about the educators’ implementation strategies, but in order to gain evidence-based knowledge about the effects of these strategies, future research designs should include a randomised intervention study with a control group and longitudinal follow-ups. A strength with respect to the credibility of the results is the authors’ previous experience in practice and research in the field of intellectual disability. Both authors participated in the data collection, interpretation, and analysis of the texts from the observations, interviews, and field notes. The analysis and interpretation were conducted both independently of one another and through a number of sessions, where our role and behaviour as researchers were also discussed. During data collection, we tried to be part of the group [35,36], sitting together with the course leaders and course members. The course leaders perceived this as positive; they commented that the sessions were calm and rewarding when one of us came to visit and that the course members welcomed such a visit.

## 5. Conclusions and Implications

The results of this healthy ageing intervention, from the perspective of the course leaders, showed that they used the implementation strategies of social togetherness and learning together to facilitate the processing of changes associated with ageing for older adults with intellectual disability, thereby contributing to a good life in old age. The course leaders facilitated learning by showing responsiveness to and respect for the course members; they met the course members’ need to socialise and talk about daily life and were then able to move on to discussions about ageing. The course leaders made every effort to create a sense of security in the social relationships between members, and the learning about ageing was facilitated by the study guides.

This education is too resource-intensive and is delivered over a long period of time, as evidenced by the changes in course leaders and course members, as well as the difficulties in recruiting people with mild intellectual disability to the intervention, despite this group being the largest compared to moderate and severe intellectual disability. One suggestion is to integrate the intervention into the regular disability service, where two experienced staff members are assigned to use the study guides developed for “Good Life in Old Age”. Preferably, the intervention should cover more than one disability service in order to save resources.

This new tailor-designed educational intervention about ageing shows good potential, but less resource-intensive interventions should also be developed and preferably integrated into the disability service. There is also a need to develop and test various models of healthy ageing interventions in future research for people with different levels of intellectual disability. Finally, there is an urgent need to evaluate the current new tailored healthy ageing intervention from the perspective of people with mild intellectual disability themselves.

## Figures and Tables

**Table 1:** Overview of the healthy ageing intervention: 50 meetings focused on ageing, accommodation, activity, and participation.

**TERM 1: 15 meetings on ageing and accommodation**	**TERM 2: 15 meetings on accommodation and activity**
1.1 General introduction to the course	2.1 Introduction—reconnecting with the theme of accommodation
1.2 Concerning ageing with dementia (from the education platform of the Swedish Dementia Centre)	2.2 In preparation for talking to decision makers
1.3 Concerning health and lifestyle (from the same platform as above)	2.3 Meeting with decision makers
1.4 What sort of accommodation do you want in old age? Wishes and dreams	2.4 Rounding off the theme of accommodation
1.5 Planning a study visit	2.5 Introduction: Concerning work and leisure; wishes and dreams
1.6 Study visit 1 (senior/55+/sheltered housing)	2.6 Planning a study visit
1.7 Sorting out impressions of the study visit	2.7 Study visit 1 (e.g., meeting place for pensioners without intellectual disability)
1.8 Planning a study visit	2.8 Sorting out impressions of the study visit
1.9 Study visit 2 (retirement home)	2.9 Planning a study visit
1.10 Sorting out impressions of the study visit	2.10 Study visit 2 (activities for persons with intellectual disability that are available where they live)
1.11 Planning a study visit	2.11 Sorting out impressions of the study visit
1.12 Study visit 3 (group accommodation provided in accordance with the Act LSS concerning Support and Service for Persons with Certain Functional Impairments, if not too far away)	2.12 Planning a study visit
1.13 Sorting out impressions of the study visit	2.13 Study visit 3 (activities arranged by pensioners’ associations)
1.14 Moving: What support is needed to ensure security and participation?	2.14 Sorting out impressions of the study visit
1.15 Summing-up of the theme of accommodation. Wishes and dreams—are they the same as before?	2.15 Summing-up of the theme of activity. Wishes and dreams—are they the same as before?
**TERM 3: 10 meetings on activity and ageing**	**TERM 4: 10 meetings on participation**
3.1 Introduction—reconnecting with the theme of activity	4.1 Introduction involving the summing-up of terms 1–3
3.2 In preparation for talking to decision makers	4.2 Ways of increasing participation: Part 1. Discussion of how much the course members influence decision making concerning the events of their own lives
3.3 Meeting with decision makers	4.3 Ways of increasing participation: Part 2. Consideration of what the course has offered when it comes to the enhancement of influence and participation
3.4 Rounding off the theme of activity	4.4 One or more guests/speakers invited in accordance with the group’s wishes; an opportunity for delving more deeply into one or more aspects of the course
3.5 Retirement: What needs thinking about? What was your own experience of it?	4.5–4.9 Optional theme: The purpose is to provide deeper knowledge and to fill any gaps in knowledge about the subjects of the educational intervention
3.6 How can one’s social network be maintained and, indeed, increased in old age?	
3.7 Concerning death and bereavement (from the education platform of the Swedish Dementia Centre)	
3.8 Thoughts on the changes that have occurred, in oneself and in society, during one’s lifetime	
3.9 Further consideration of the theme of accommodation	
3.10 Further consideration of the theme of ageing (from the education platform of the Swedish Dementia Centre and from booklets of the Swedish National Association for People with Intellectual Disability). Summary and rounding off the term	4.10 Conclusion and evaluation—Information about what comes next: Evaluation, the ceremonious presentation of diplomas, and the festive consumption of food and drink

**Table 2:** Guidelines for the observations and ad hoc interviews with the course leaders.

**The researcher observed and wrote field notes after each course session based on the following aspects:**
(1) What is the subject of this course session, and how is it conducted?
(2) How do the course members listen? (Level of attention, which strategies facilitate concentrated listening and learning, disturbing elements of the situation)
(3) How does the leader listen and meet the course members’ needs? (Does the leader listen to questions and signals from the course members?)
(4) Who is asking questions, and what kinds of questions? (Is the question related to the topic of the day? Is it a question or a suggestion?)
(5) How does communication between the course members and the course leader take place? (Does the course leader encourage questions and how? How are questions answered? Who gets involved in the communication?)
(6) How does the group interact? (Is everyone allowed to speak in the group? How do the course members communicate? Do they assign different roles to one another?)
**Ad hoc interviews with the course leader immediately after the observation:**
The course leaders were interviewed individually about their experiences of how the course members with intellectual disability were able to absorb the content, how successful they were in communicating with the course members, and the interaction in the group. The questions asked by the researcher were related to learning strategies and were formulated on the basis of the specific course session.

**Table 3:** Overview of the findings from the healthy ageing intervention in terms of themes and sub-themes.

Themes	Sub-Themes
Promoting social togetherness through everyone’s participation	Respectful meetings in close-knit groups Reinforcing social coherence
Learning together and from each other through recognition and consolidation	Responsiveness is the path to knowledge The right to be heard and to have an influence Facilitated responsibility for knowledge acquisition

## Data Availability

The datasets used and analysed in this study are available upon written request from the responsible researcher (G.A.), in accordance with the ethical approval guidelines.
